# Dual inhibition of the PI3K/AKT/mTOR pathway suppresses the growth of leiomyosarcomas but leads to ERK activation through mTORC2: biological and clinical implications

**DOI:** 10.18632/oncotarget.13987

**Published:** 2016-12-16

**Authors:** Benjamin Fourneaux, Vanessa Chaire, Carlo Lucchesi, Marie Karanian, Raphael Pineau, Audrey Laroche-Clary, Antoine Italiano

**Affiliations:** ^1^ Université de Bordeaux, Bordeaux, France; ^2^ Institut National de la Santé et de la Recherche Medicale (INSERM), Institut Bergonié, Bordeaux, France; ^3^ Department of Medical Oncology, Institut Bergonié, Bordeaux, France

**Keywords:** leiomyosarcomas, PI3K/AKT/mTOR pathway, BEZ235, BKM120, everolimus

## Abstract

The PI3K/AKT/mTOR pathway plays a crucial role in the development of leiomyosarcomas (LMSs). In this study, we tested the efficacy of dual PI3K/mTOR (BEZ235), PI3K (BKM120) and mTOR (everolimus) inhibitors in three human LMS cell lines. *In vitro* and *in vivo* studies using LMS cell lines showed that BEZ235 has a significantly higher anti-tumor effect than either BKM120 or everolimus, resulting in a greater reduction in tumor growth and more pronounced inhibitory effects on mitotic activity and PI3K/AKT/mTOR signaling. Strikingly, BEZ235 but neither BKM120 nor everolimus markedly enhanced the ERK pathway. This effect was reproduced by the combination of BKM120 and everolimus, suggesting the involvement of mTORC2 via a PI3K-independent mechanism. Silencing of RICTOR in LMS cells confirmed the role of mTORC2 in the regulation of ERK activity. Combined treatment with BEZ235 and GSK1120212, a potent MEK inhibitor, resulted in synergistic growth inhibition and apoptosis induction *in vitro* and *in vivo*. These findings document for the first time that dual PI3K/mTOR inhibition in leiomyosarcomas suppress a negative feedback loop mediated by mTORC2, leading to enhanced ERK pathway activity. Thus, combining a dual PI3K/mTOR inhibitor with MEK inhibitors may be a relevant approach to increase anti-tumor activity and prevent drug resistance in patients with LMS.

## INTRODUCTION

Leiomyosarcomas (LMSs) are an uncommon group of malignant tumors composed of cells that show distinct smooth muscle differentiation and that represent 15–20% of all soft-tissue sarcomas, making LMS one of the most frequent sarcoma subtypes [1]. The current interventions for leiomyosarcoma include surgical excision, radiotherapy and chemotherapy with an aggressive approach taken for high-grade tumors [2]. However, the prognosis is poor, with up to 40% of patients experiencing metastatic relapse despite optimal locoregional treatment [3, 4]. Further options for the treatment of LMSs are needed.

From a genetic point of view, LMSs are characterized by numerous genomic alterations, which include multiple regions of genomic amplifications and deletions [5–9]. Among these regions, a large region of deletion on chromosome 10 encompassing PTEN (phosphatase and tensin homolog), a tumor suppressor gene and negative regulator of phosphoinositide-3-kinase (PI3K), is one of the most frequent aberrations (40–50% of cases). Moreover, *in vitro* and *in vivo* studies have highlighted the critical role of the PI3K/mTOR pathway in smooth muscle transformation and LMS development [10]. In these studies, mTOR inhibition was associated with significant anti-tumor activity [11]. These data have been recently confirmed in the clinical setting by a pilot study of patients with advanced leiomyosarcoma who were treated with temsirolimus with significant benefit [12]. Moreover, immunohistochemical evaluation of the downstream target of mTOR, phosphorylated S6 ribosomal protein (p-S6RP), has been correlated with an early clinical response to mTOR inhibitors (AP23573) administered either alone or in combination to a cohort of patients with varying types of sarcomas [13]. However, several studies have shown that inhibition of mTOR by rapamycin and its analogs is associated with a loss of negative feedback control of the MAPK pathway [14] and PI3K/AKT/mTOR pathway in solid tumors [15, 16]. This finding may explain the transient benefit observed with mTOR inhibitors in a clinical setting and the need for more potent strategies to target this pathway [17].

PI3K and mTOR both belong to the PI3K-related kinase superfamily and share structural domains. Consequently, certain inhibitory compounds target both kinases [18]. Dual inhibitors of PI3K and mTOR target the active sites of both holoenzymes to inhibit the pathway both upstream and downstream of AKT, thus avoiding the problem of AKT activation following abolition of the mTORC1-S6K-IRS-1 or S6K-mTORC2-AKT negative feedback loops. This aberrant activation is known to occur with rapalogs such as sirolimus, everolimus and temsirolimus [15, 16, 19]. Moreover, a recent pre-clinical study has shown favorable selective activity of these inhibitors in LMS cell lines [20]. Here, we report an original study investigating the effects of dual inhibition of PI3K and mTOR in human leiomyosarcomas on anti-tumor activity, especially the biological consequences on components of the PI3K/AKT/mTOR and RAS/MEK/ERK pathways.

## RESULTS

### PI3K/AKT/mTOR pathway inhibitors inhibited proliferation and caused apoptosis in LMS cell lines

**Figure 1:**
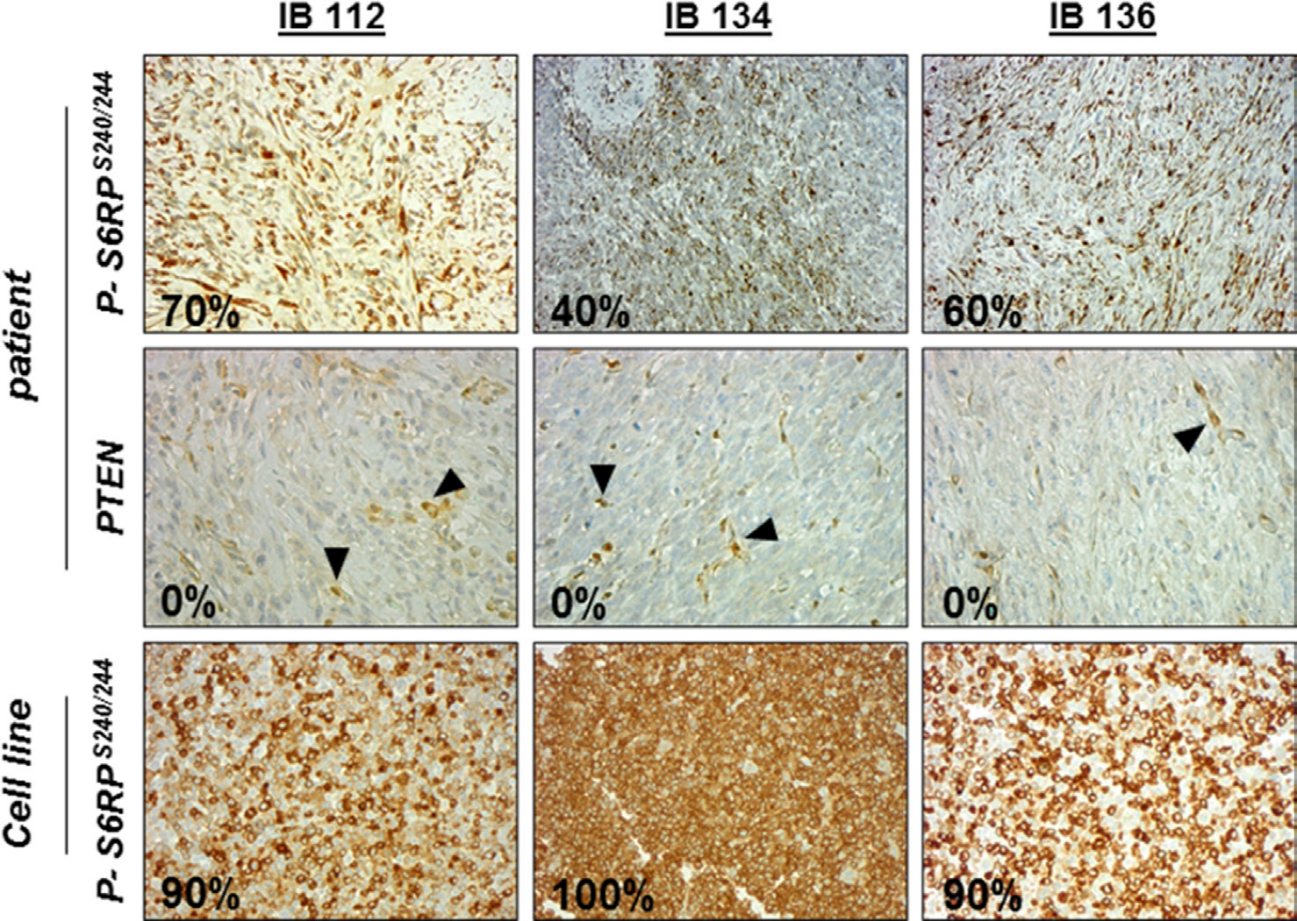
Immunohistochemical (IHC) staining against p-S6RPser240/244 and PTEN in leiomyosarcoma (LMS) disease tissues and cell lines Immunohistochemical staining pictures of LMS tissue samples with anti-p-S6RP^ser240/244^ and anti-PTEN antibodies and of cell line pellets with anti-p-S6RP^ser240/244^. Endothelial cells (positive control) are indicated by black arrows.

**Figure 2:**
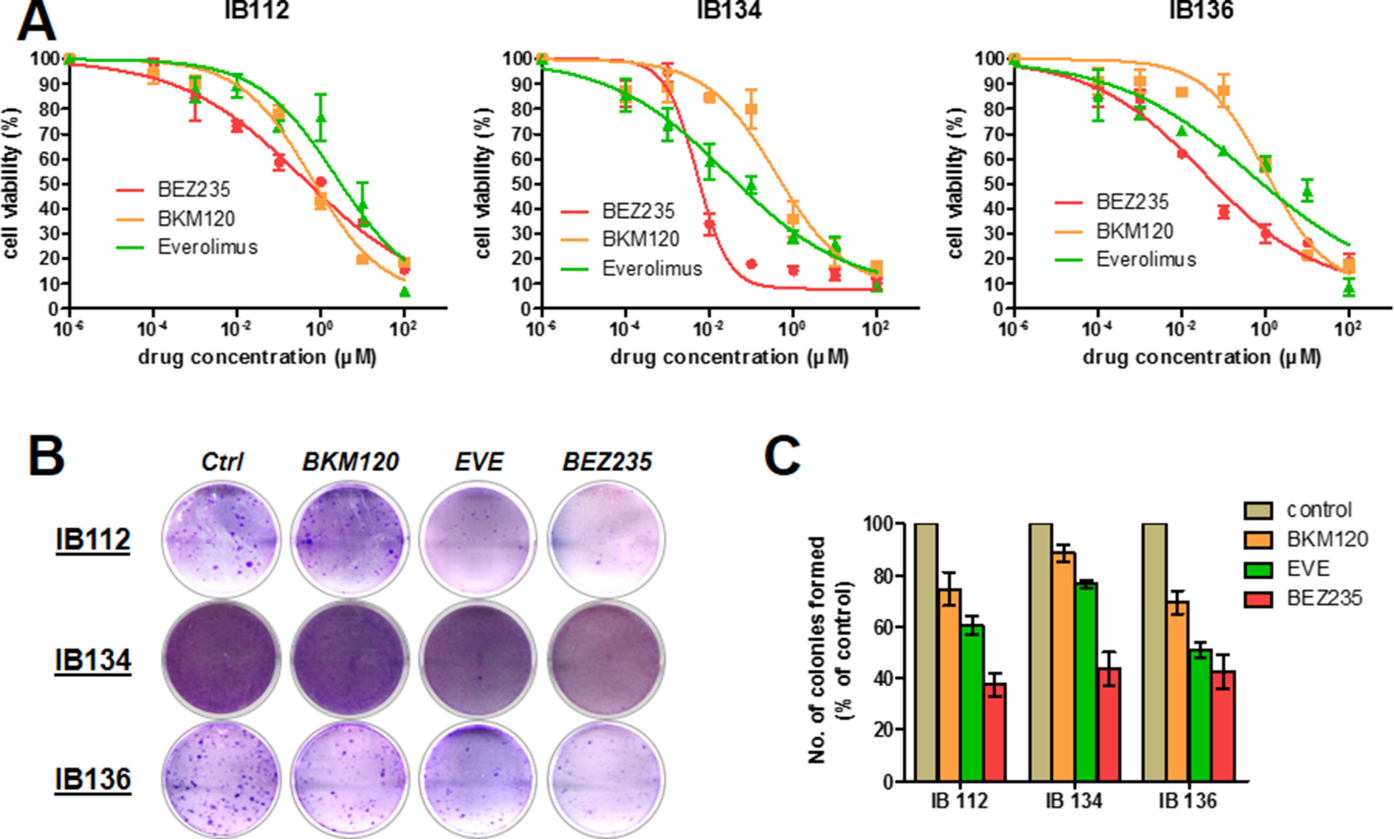
Antiproliferative and apoptotic activities of BEZ235, BKM120 and everolimus (EVE) in LMS cell lines Growth curves indicating growth inhibition of the 3 LMS cell lines (IB112, 134 and 136) after treatment for 72 hours (**A**). Representative pictures of the clone formation assay for cell lines treated for 72 hours by PI3K/mTOR pathway inhibitors at their IC_50_ value (**B**). The average percentage of monoclonal cell number for each cell line (**C**).

**Figure 3:**
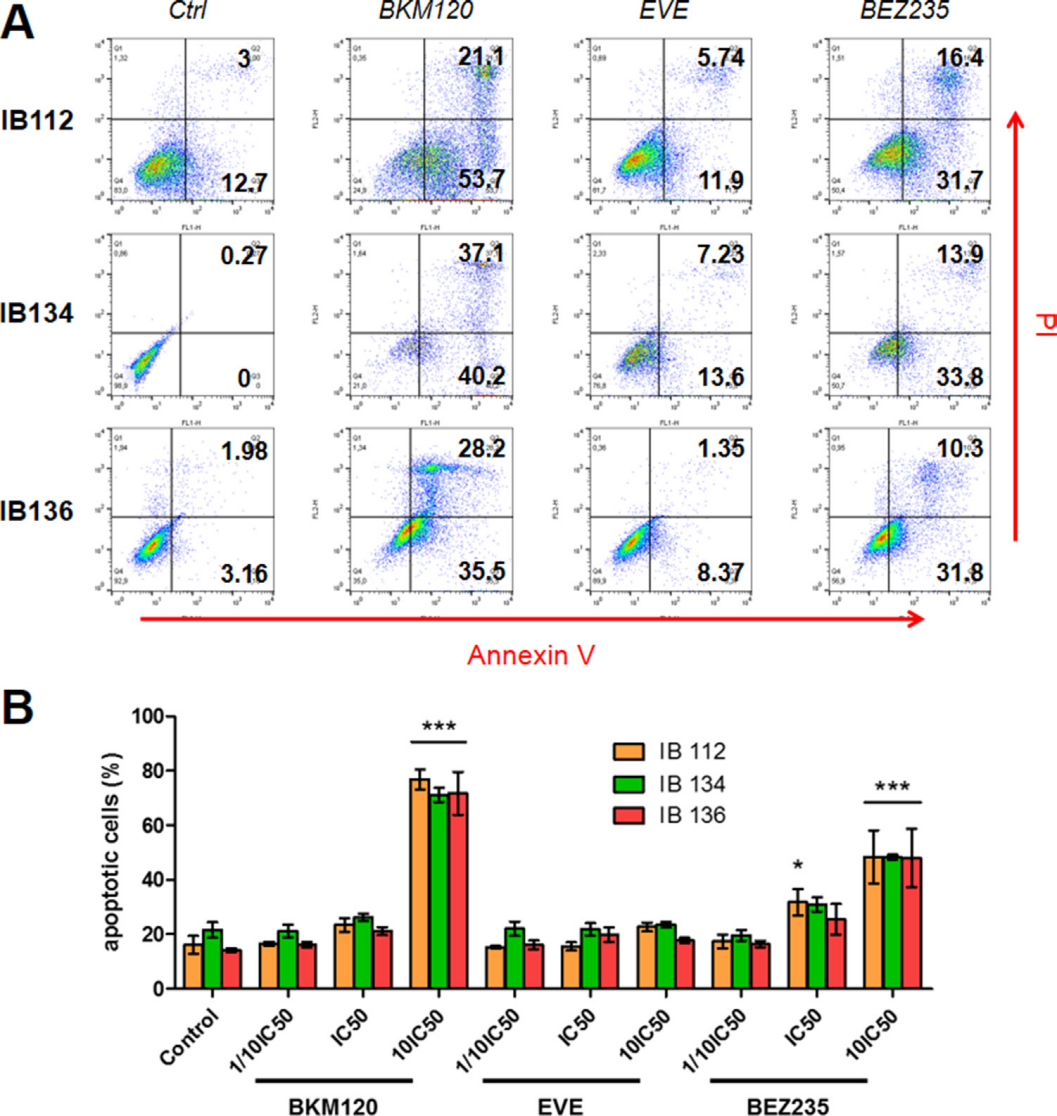
Effect of BEZ235, BKM120 and everolimus (EVE) on LMS cell apoptosis Representative dot-plot diagrams of flow cytometry with annexin V/PI for LMS cells treated for 72 hours with BEZ235, BKM120 and EVE at 10-fold IC_50_ values (**A**). Percentage of apoptotic cells after treatment for 72 hours with 3 drug concentrations (1/10 IC_50_, IC_50_ value, 10-fold IC_50_) (**B**). Data are presented as the mean ± SEM of three independent experiments. *, drug *p* < 0.05 vs. control; ***, drug *p* < 0.001 vs. control (two-way ANOVA).

For the purposes of this study, we used three LMS cell lines derived from surgical specimens obtained from consenting patients. All patient tumors displayed the loss of PTEN expression and strong p-S6RP^S240/244^ staining indicating sustained overactivation of the PI3K/AKT/mTOR pathway (Figure 1). LMS cells derived from patient tumors showed similar p-S6RP^S240/244^ staining, but in the absence of endothelial cells in the LMS cell line pellets, interpretation of PTEN staining could not be performed (Figure 1). We assessed the respective anti-tumor activity of the following PI3K/AKT/mTOR pathway inhibitors: BEZ235 (dual inhibitor of PI3K, mTORC1 and mTORC2), BKM120 (PI3K inhibitor) and everolimus (mTORC1 inhibitor). We observed dose-dependent growth suppression that was more strongly induced in all cell lines by BEZ235 (IC_50_ range, 0.001 to 0.1 μM) than by either BKM120 or everolimus (range, 0.01 to 1.6 μM; Figure 2A). Additionally, after treatment with the respective IC_50_ values of inhibitors for 72 hours, all leiomyosarcoma cell lines exhibited a significant decrease in colony formation in the clonogenic assays upon exposure to BEZ235 compared with either BKM120 or everolimus (Figure 2B). Compared to cells with untreated medium (control), colony formation by IB112, IB134 and IB136 cells was reduced approximately 60% after treatment with BEZ235 at its IC_50_ value (Figure 2C), while cells exhibited a range of 10–20% (with BKM120) and 30–45% (with everolimus) inhibition of colony formation (Figure 2C). Interestingly, we failed to detect any significant induction of apoptosis in LMS cells with PI3K/AKT/mTOR pathway inhibitors at the same concentration (Figure 3B). Only exposure to high doses of BEZ235 and BKM120 led to induction of apoptosis as revealed by 40% and 65% increases in the percentage of annexin V- and PI-positive cells compared to control cells (Figure 3A and 3B). No effect was observed with everolimus.

### BEZ235 induced ERK pathway activation

**Figure 4:**
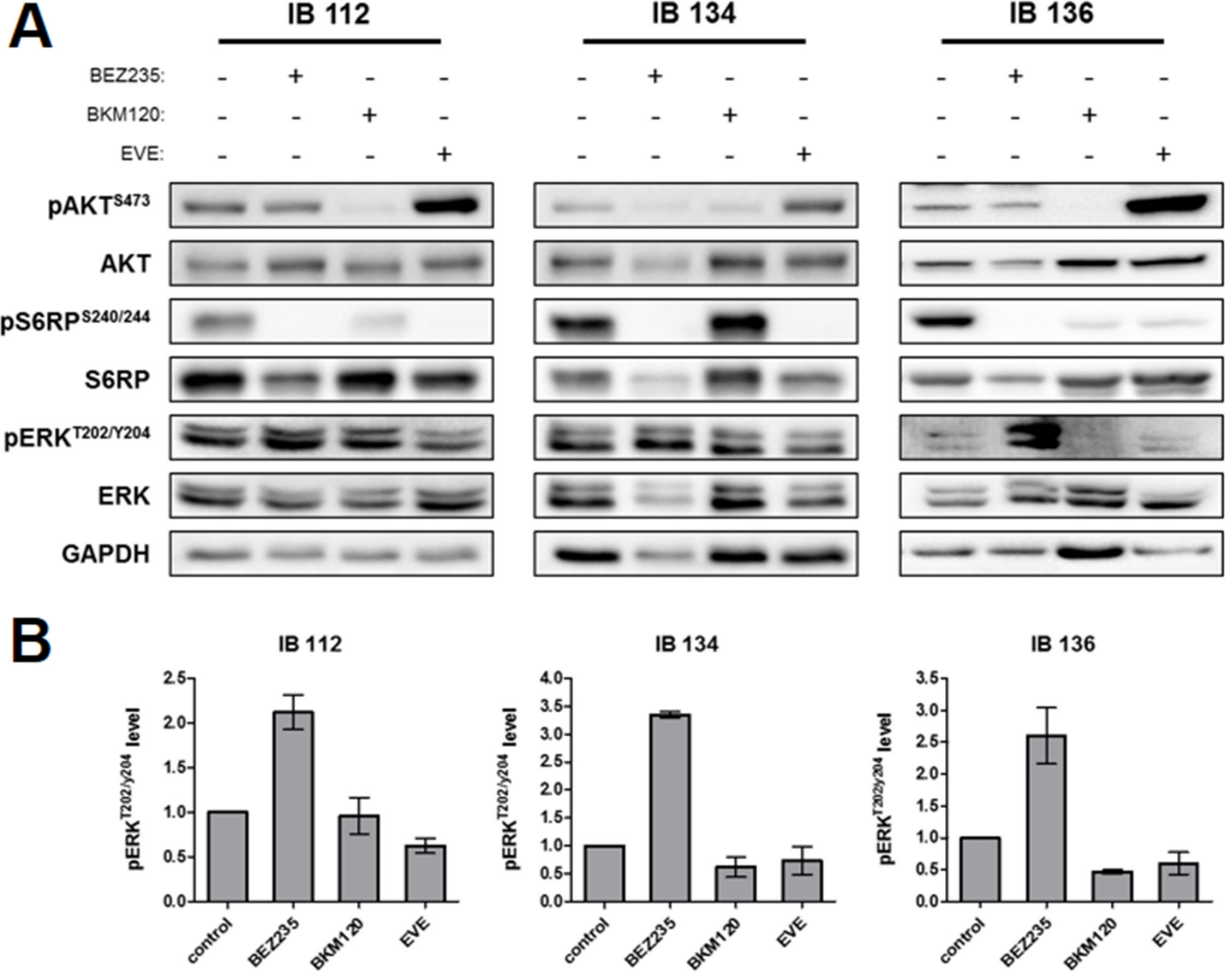
Downstream kinase inhibition by BEZ235, BKM120 and everolimus (EVE) in LMS cell lines Representative western blotting of active kinase and total kinase levels of the PI3K/mTOR and MAPK pathway with GAPDH as a loading control (**A**). Cells were treated for 72 hours at the IC_50_ value of each drug. Representative signal intensities for p-ERK1/2^thr202/tyr204^ were normalized to those for total ERK in each cell line (**B**). Data are presented as the mean ± SEM of three independent experiments.

We further explored the capacity of these drugs to inhibit downstream kinases of the PI3K/AKT/mTOR pathway by western blotting using phosphorylation of both AKT and S6RP as a marker of pathway activation. Consistent with the proliferation results, BEZ235 was most effective in inhibiting p-S6RP^S240/244^ in the 3 LMS cell lines. This observation indicated a more complete blockade that was quickly achieved and sustained with dual PI3K/mTOR inhibition (Figure 4A). As expected and described in several studies, everolimus enhanced p-AKT^S473^ accumulation consequent to suppression of the S6K-mTORC2-AKT feedback loop [19]. Strikingly, BEZ235 markedly induced ERK activation, while this effect was not observed with either BKM120 or everolimus (Figure 4A and 4B). However, we wondered whether the combination of BKM120 and everolimus could reproduce the accumulation of p-ERK^T202/Y204^ observed with BEZ235 treatment. Synergy between BKM120 and everolimus was observed on LMS cell line growth, with median combination indices of 0.62 for IB112, 0.1 for IB134 and 0.1 for IB136 (Supplementary Figure S1A), and on apoptosis induction only for the IB136 cell line, with an increase in the percentage of annexin V- and PI-positive cells (30%) compared with monotherapy (20% for BKM120 and 13% for everolimus; Supplementary Figure S1B and S1C). Interestingly, dual inhibition of PI3K and mTORC1 by the combination of BKM120 and everolimus mimicked the effects of BEZ235 to induce MAPK pathway activation (Supplementary Figure S1D). Considering the similar effect on MAPK pathway activation observed with BEZ235, we hypothesized that p-ERK^T202/Y204^ accumulation could be caused by a feedback mechanism implicating RICTOR, a scaffold protein from the mTORC2 complex that is known to be regulated by S6K1 [19].

### Leiomyosarcoma xenografts were more sensitive to dual PI3K/mTOR inhibition than PI3K or mTORC1 inhibition alone

**Figure 5:**
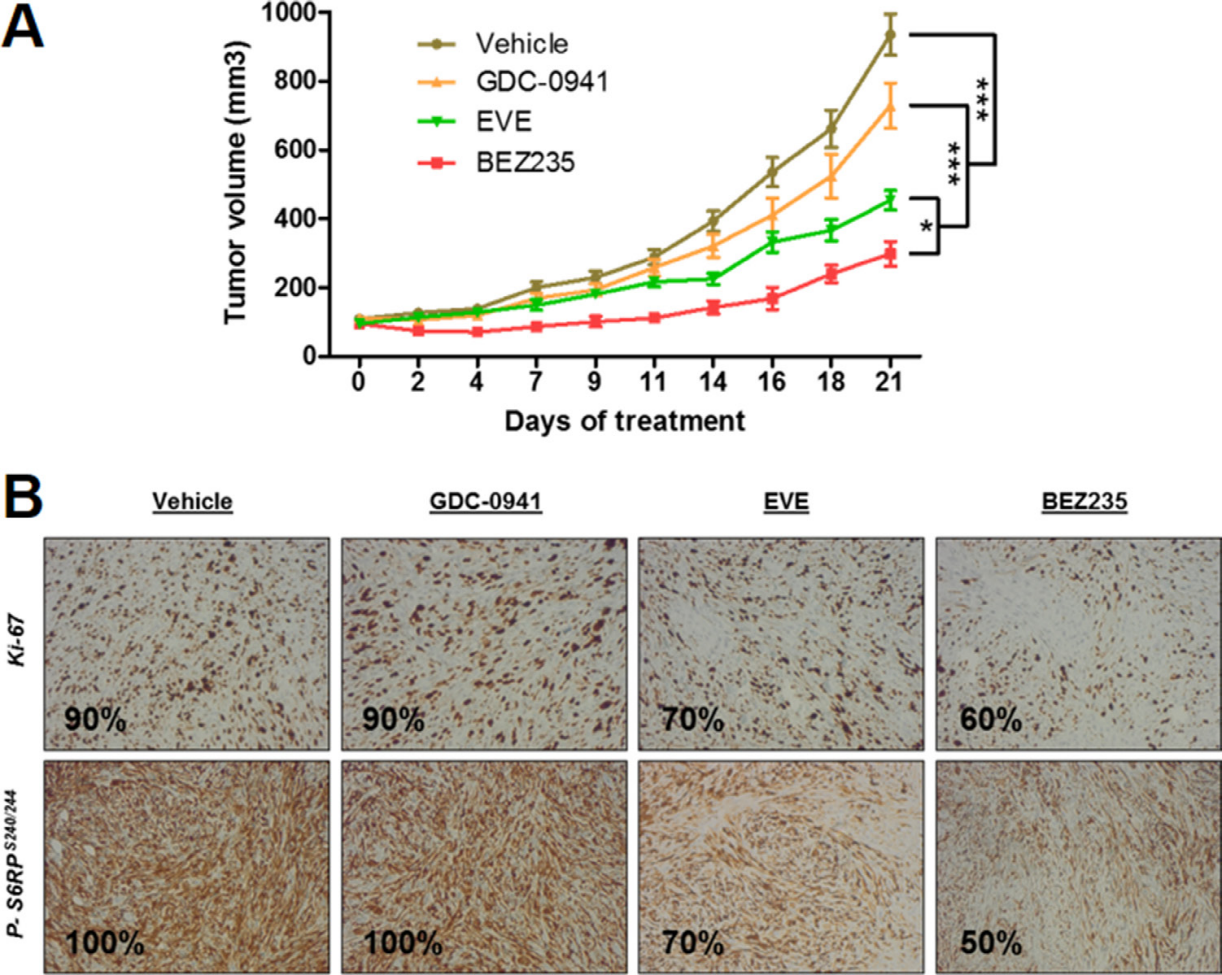
Anti-tumor effect of BEZ235, BKM120 and everolimus (EVE) on human IB136 cell xenografts in Ragγ2C−/− mice Curves of tumor volume progression during 3 weeks of treatment (**A**) Mice were randomly assigned to receive 40 mg/kg BEZ235, 50 mg/kg GDC-0941, 5 mg/kg everolimus or vehicle. The data points represent an average from 8 mice (bars, SEM). *p < 0.05; ***p < 0.001, two-way ANOVA. Immunohistochemical staining images of tumor samples treated with anti-p-S6RP^ser240/244^ and anti-Ki-67 antibodies (**B**).

These *in vitro* findings prompted us to examine the effect of PI3K/AKT/mTOR pathway inhibitors on LMS tumor growth. For the *in vivo* study, BKM120 was replaced with GDC-0941, another PI3K inhibitor. IB136 xenografts were established and grew to a size of 100 mm^3^, after which either vehicle or drugs were given 5 days a week for 3 weeks. The growth suppression induced by BEZ235 was more significant in LMS xenografts (Figure 5A). The average inhibition tumor growth (ITG) of tumors from the BEZ-treated animals was 65%, whereas those from the GDC-0941 and everolimus groups were 27% (*p <* 0.05) and 54% (*p <* 0.05), respectively, compared to the control group (*p <* 0.05). No apparent toxicity events were observed in the drug-treated animals. There were no significant changes in animal weight (data not shown). The number of tumor cells positive for Ki-67, a cell proliferation marker, was substantially lower in tumors treated with BEZ235 compared with control tumors (Figure 5B). With regard to the cellular pathway involved, as shown in the representative immunohistochemistry images, tumor xenografts from the BEZ235-treated group showed a marked decrease in the number of p-S6RP^S240/244^-positive cells compared with the other groups. Therefore, these pharmacodynamic assessments confirmed the *in vitro* activity of the drugs in terms of proliferation and pathway inhibition.

### Extinction of RICTOR-enhanced ERK activation

**Figure 6:**
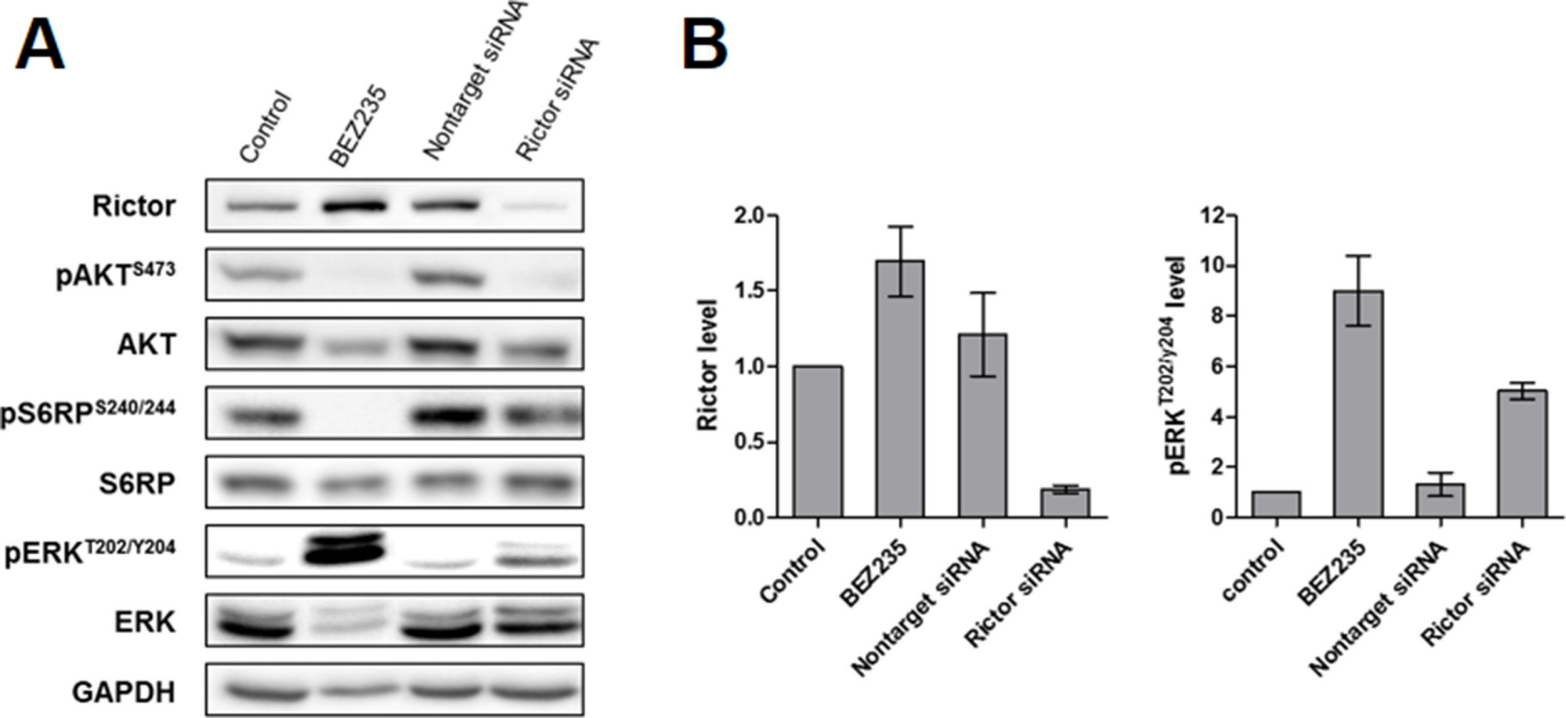
Effect of RICTOR silencing on the PI3K and MAPK downstream signaling pathways in the IB134 cell line Representative western blotting of active kinase and total kinase levels of the PI3K/mTOR and MAPK pathway with GAPDH as a loading control (**A**). LMS cell lines were incubated with BEZ235 and RNAi against RICTOR for 72 hours. Representation of the signal intensities for RICTOR and p-ERK1/2^thr202/tyr204^ were normalized to those for GAPDH and ERK, respectively (**B**). Data are presented as the mean ± SEM of two independent experiments.

To understand whether mTORC2 was involved in ERK pathway activation, we used RNAi to silence RICTOR, an essential and specific component of mTORC2. As expected, RNAi did not alter the kinase expression of AKT and S6RP and resulted in a 50–90% decrease of RICTOR protein expression levels compared to non-targeting RNAi in LMS cell lines (Figure 6A and quantification in Figure 6B for IB134; Supplementary Figure S2 for IB112 and IB136). Additionally, silencing of RICTOR did not disrupt mTORC1/S6K activity as scored by p-S6RP^S240/244^ but abolished AKT phosphorylation on Ser^473^, a modification mediated by mTORC2. Interestingly, in RICTOR-silenced LMS cells, we showed p-ERK^T202/Y204^accumulation similar to the enhancement caused by BEZ235 treatment (Figure 6A and quantification in Figure 6B for IB134; Supplementary Figure S2 for IB112 and IB136). Transfection with RNAi (AM16708-39500, Life Technologies, Carlsbad, CA, USA) targeting a different region of RICTOR mRNA also resulted in accumulated p-ERK^T202/Y204^ (data not shown). Thus, these results confirmed our hypothesis that mTORC2 is involved in MAPK pathway activation.

### GSK1120212 potentiated apoptosis induction and the anti-tumor effect of BEZ235

**Figure 7:**
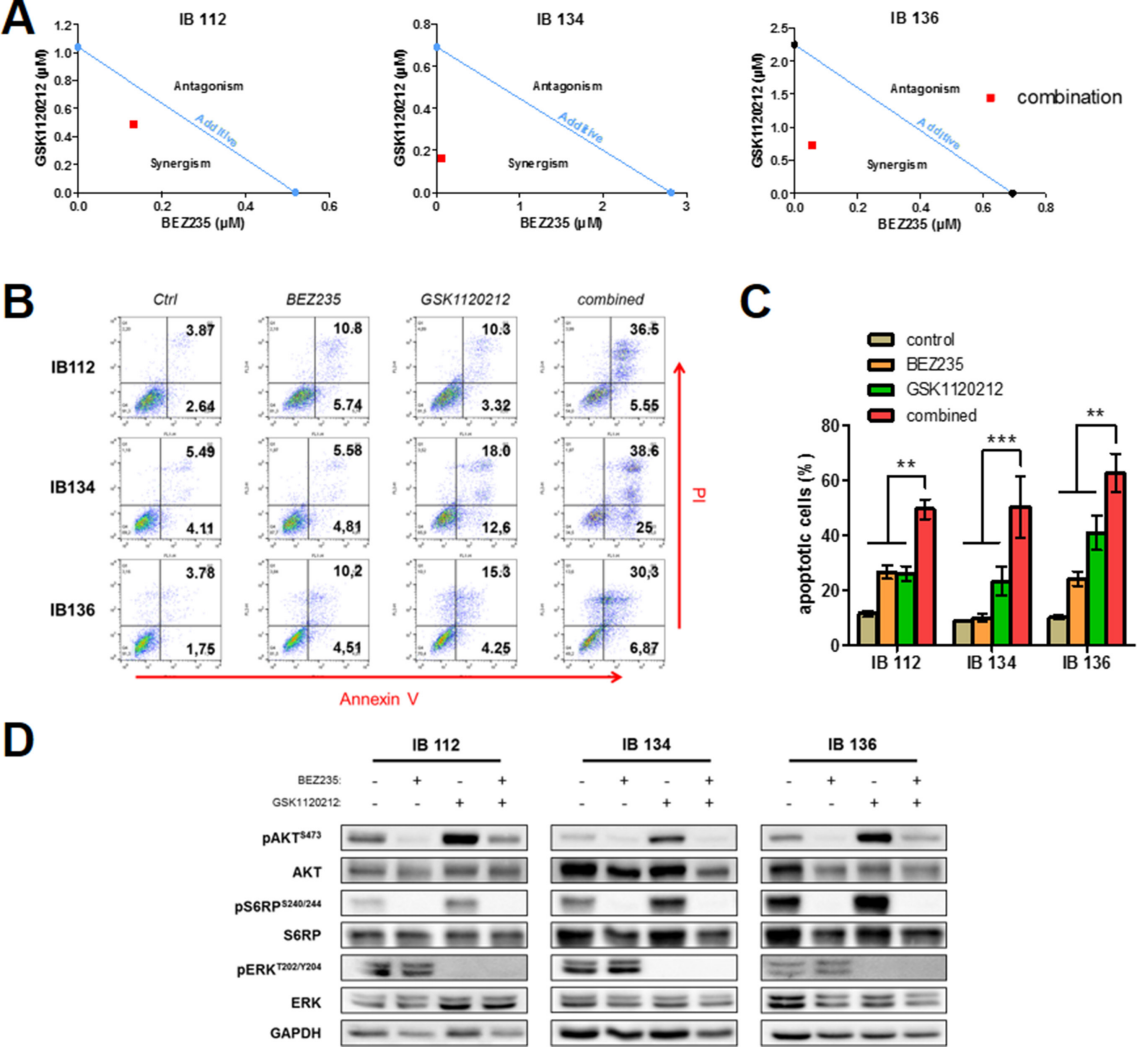
Synergistic activity of the BEZ235 and GSK1120212 combination in IB112, 134 and 136 LMS cell lines Isobologram analysis of the combination of BEZ235 and GSK1120212 in LMS cells (**A**). Combination index (CI) values for each cell line were calculated using the method developed by Chou and Talalay and are represented by a red point. Representative dot-plot diagrams of the flow cytometry results with annexin V/PI for LMS cells treated with BEZ235 and GSK1120212 either alone or in combination (**B**). Percentage of apoptotic cells after respective treatments (**C**). Immunoblotting analysis of active kinase and total kinase levels of the PI3K/mTOR and MAPK pathway with GAPDH as a loading control (**D**). Cells were treated for 72 hours at the IC_50_ value of each drug. Data are presented as the mean ± SEM of three independent experiments. **p < 0.01; ***p < 0.001, two-way ANOVA.

We showed that BEZ235 induced an accumulation of p-ERK^T202/Y204^ protein, a marker of MAPK pathway activation. The crosstalk between the PI3K and MAPK pathways has long been known. In fact, PI3K and mTOR blockade may result in the activation of compensatory pathways of MAPK that could potentially reduce the anti-tumor effects of PI3K/mTOR inhibitors [21]. Thus, we decided to further investigate the effects of combined targeting of the MAPK and PI3K/AKT/mTOR signaling cascades in leiomyosarcoma cells. To this end, we administered a diagonal constant ratio combination design of BEZ235 and GSK1120212, a potent MEK inhibitor, in LMS cell lines according to the Chou and Talalay proposition [22]. *In vitro* synergy was observed with BEZ235 in association with GSK1120212 on the growth of LMS cell lines with median combination indices of 0.72 for IB112, 0.25 for IB134 and 0.4 for IB136 (Figure 7A) and on apoptosis induction with an increase in the percentage of annexin V- and PI-positive cells (range of apoptotic cells, 44–66%) compared with either drug alone (range of apoptotic cells, 10–27% for BEZ235 and 23–41% for GSK1120212) as shown in Figure 7B and 7C. Additionally, after exposure at the IC_50_ value for 72 hours, GSK1120212 was effective in inhibiting p-ERK^T202/Y204^ in all LMS cell lines, and as expected, due to presence of crosstalk between the PI3K and MAPK pathways, we observed p-AKT^S473^ accumulation consequent to the suppression of MAPK pathway activity (Figure 7C) [16]. This treatment combined with BEZ235 prevented ERK and AKT over-activation induced by BEZ235 and GSK1120212 alone, respectively (Figure 7C).

**Figure 8:**
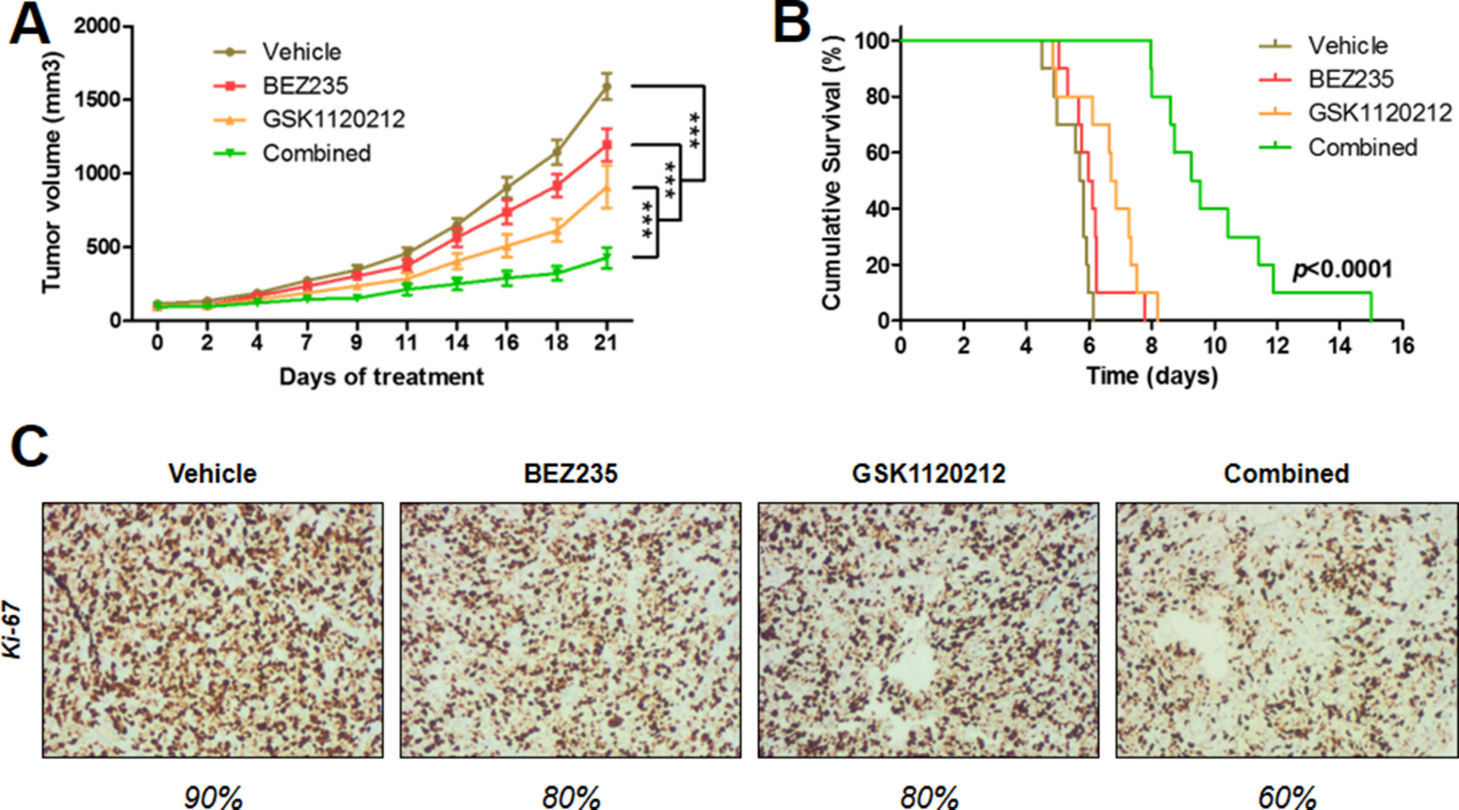
Synergistic anti-tumor effect of the BEZ235 and GSK1120212 combination in human IB136 cell xenografts in Ragγ2C−/− mice Curves of tumor volume progression during 3 weeks of treatment (**A**). Mice were randomly treated with vehicle, 10 mg/kg BEZ235, 0.5 mg/kg GSK1120212 or a combination of both drugs. The data points represent an average from 10 mice (bars, SEM). ***p < 0.001, two-way ANOVA. Kaplan-Meier curves for tumor doubling times (**B**). Immunohistochemical staining images of tumor samples treated with the anti-Ki-67 antibody (**C**).

Moreover, we tested whether the MAPK pathway blockade could enhance the anti-tumoral effect of the dual PI3K/mTOR inhibitor *in vivo*. IB136 xenografts were established and grew to a size of 100 mm^3^, after which either vehicle or drugs were given 5 days a week for 3 weeks. The BEZ235 and GSK1120212 combination group showed a stronger anti-tumor effect with a significant (*p <* 0.001) reduction of tumor growth (average tumor volume at endpoint, 428.5 ± 70.2 mm^3^) compared with either drug alone (1193.9 ± 112.1 mm^3^ for BEZ235 and 910.5 ± 143.1 mm^3^ for GSK1120212) or vehicle (1591 ± 90.8 mm^3^) as shown in Figure 8A. Also, the survival of mice (from the first day of the experiment until the day when the tumor size doubled) showed that the combination treatment highly significantly slowed the rate of tumor growth (9.4 days for median survival) compared to the control (5.8 days) and to the individual drug (6.4 days) treatment groups (*p <* 0.0001) as shown in Figure 8B. No apparent toxicity events were observed in the drug-treated animals. There were no significant changes in animal weight (data not shown). Further analyses by immunohistochemical experiments showed that the number of tumor cells positive for Ki-67 was substantially less in LMS tumors treated with the combination treatment compared with tumors treated with either drug alone or control tumors (Figure 8C).

## DISCUSSION

We report an original study assessing the respective *in vitro* and *in vivo* effects of a drug panel targeting different components of the PI3K/AKT/mTOR pathway on human leiomyosarcoma, an extremely rare form of cancer. This pathway is one of the most frequently dysregulated signaling cascades in cancer [23]. One challenge related to the development of anti-cancer drugs targeting this signaling cascade is to identify agents capable of achieving sufficiently strong inhibition of the pathway (and the subsequent anti-tumor activity). Dual inhibitors of PI3K and mTOR, such as BEZ235, target the active sites of both holoenzymes, inhibiting the pathway both upstream and downstream of AKT and avoiding the problem of AKT activation following the abolition of the mTORC1-S6K-IRS-1 negative feedback loop [24]. The loss of this feedback loop is known to occur with rapalogs, the first PI3K pathway-targeted agents approved for the treatment of cancer. Dual inhibitors are also expected to be more active than pan-PI3K inhibitors, particularly in tumors with alterations downstream of PI3K and upstream of mTOR (e.g., PTEN or TSC1/2), such as LMS [25]. We have demonstrated that dual inhibition of PI3K and mTOR is associated with strong anti-tumor activity in LMS, which was significantly higher than that of either mTOR inhibition (everolimus) or PI3K inhibition (BKM120) alone. Viability changes were reflected in the apoptotic effects produced by BEZ235, while apoptosis was not observed with everolimus, indicating that PI3K is sufficient for cell survival.

Another important challenge for the development of drugs targeting the PI3K/AKT/mTOR pathway is the elucidation of acquired resistance mechanisms that either restore PI3K signaling or activate parallel pathways in the presence of inhibitors. The existence of redundancy and feedback loops between the RAS-RAF-MEK-ERK and PI3K-AKT-mTOR signaling networks is undisputed [26]. mTOR nucleates two distinct multi-protein complexes, the mTOR complex 1 (mTORC1) and mTOR complex 2 (mTORC2). In contrast to mTORC1, for which many upstream signals and cellular functions have been defined, relatively little is known about mTORC2 biology. Several studies have shown that mTORC2 plays key roles in various biological processes including cell survival, metabolism, proliferation and cytoskeletal organization [27]. It has been shown that allosteric mTORC1 inhibitors such as everolimus can lead to activation of the ERK pathway as a result of a PI3K-dependent mechanism, thus adding a new level of complexity to the previously described negative feedback loop involving mTORC1/PI3K/AKT [14]. We found that the dual PI3K/mTOR inhibitor BEZ235 profoundly inhibits mTORC1, mTORC2, and PI3K but induces overactivation of the RAS/MEK/ERK pathway in LMS cells. To understand the mechanism by which dual PI3K/mTOR inhibitors such as BEZ235 promoted ERK activation, we determined the role of the previously described feedback loop involving mTORC1/S6K/PI3K/ERK in response to rapamycin analogs [15, 16]. Neither mTORC1 inhibition (everolimus) nor PI3K inhibition (BKM120) at concentrations that completely blocked the mTORC1/S6K axis produced any detectable enhancement of ERK activation in LMS cells, suggesting a PI3K-independent mechanism. Strikingly, we observed that the combination of BKM120 and everolimus induced MAPK pathway activation similar to BEZ235, raising the possibility that this activation was the result of the suppression of a negative feedback loop involving mTORC2. Indeed, S6K1 (activated by mTORC1) and TSC1/2 (inhibited by PI3K/AKT) negatively and positively regulate mTORC2, respectively [28]. To confirm this hypothesis, we used RNAi to silence RICTOR, a scaffold protein in the mTORC2 complex. We observed that knockdown of RICTOR increased the baseline levels of ERK phosphorylation similar to BEZ235 treatment (Figure 6 and Supplementary Figure S2). Therefore, we showed that dual inhibition of PI3K/mTOR suppresses a PI3K-independent feedback loop involving mTORC2 and results in MAPK pathway activation in LMS cells. Interestingly, an identical finding was reported in a pancreatic cancer model, suggesting a common effect for different malignancies [29].

Given the overactivation of the RAS/MEK/ERK pathway induced by BEZ235, we investigated whether this effect could counterbalance the growth-suppressive action of dual mTOR/PI3K inhibition in LMS cells. We found that the use of the MEK inhibitor GSK1120212 abrogated the effect of BEZ235 on p-ERK^T202/Y204^ accumulation, resulting in a synergistic increase in LMS cell apoptosis and significantly higher inhibition of tumor growth. Thus, in addition to the cytostatic effect, this multidrug combination exerted cytotoxic effects (Figure 7). This result is in agreement with that from Soares *et al*., who showed that MEK1/2 inhibition suppressed the enhanced ERK activation induced by PI3K/mTOR dual inhibitors and enhanced the effects of growth inhibition due to BEZ235 in pancreatic cancer cells. Importantly, we were able to confirm our *in vitro* findings *in vivo* with a statistically significant greater anti-tumor effect of the BEZ235 and GSK1120212 combination versus the same agents used as monotherapies (Figure 8).

There are few examples of the successful use of targeted inhibitors to disrupt the aberrant signaling pathways that malignant mesenchymal tumors require for continued growth. These examples include imatinib-mediated inhibition of c-KIT in gastrointestinal stromal tumors [30], inhibition of PDGFR-β (platelet-derived growth factor receptor) by imatinib in dermatofibrosarcoma protuberans [31], mTOR inhibition in perivascular epithelioid cell tumors [32] and EZH2 (enhancer of zeste homolog 2) inhibition in INI1-or SMARCA4-deficient sarcomas [33]. Our pre-clinical results suggest that dual targeting of the PI3K/AKT/mTOR pathway in combination with MAPK inhibition is a promising therapeutic strategy against leiomyosarcoma.

## MATERIALS AND METHODS

### Cell lines

The cell lines used in this study were provided by the Biological Resources Center of Institut Bergonié (CRB-IB). Experiments were performed in accordance with the French Public Health Code (articles L. 1243-4 and R. 1243-61). The soft tissue leiomyosarcoma cell lines IB112 and IB136 and the uterine leiomyosarcoma cell line IB134 were derived from human surgical STS (soft-tissue sarcoma) specimens after obtaining patient consent and were established as previously described [34]. Authentication of the cell lines was performed using aCGH by comparing the cell line with the corresponding originating tumor. All cell lines were cultured in RPMI 1640 medium, GlutaMAX^TM^ Supplement (Sigma-Aldrich Life Technologies, Saint Louis, MO, USA) supplemented with 10% (v/v) fetal bovine serum (FBS), 1% penicillin/streptomycin, and 0.2% Normocin (InvivoGen, Toulouse, France) at 37°C in an environment containing 5% CO_2_. Cells were routinely passaged every 2 to 3 days.

### Inhibitor treatments

BEZ235 (dual PI3K/mTOR inhibitor), BKM120 (PI3K inhibitor), everolimus (mTOR inhibitor) and GSK1120212 (MEK inhibitor) were purchased from Selleck Chemicals (Houston, TX, USA) and were prepared as 2.1 mmol/L, 24 mmol/L, 10 mmol/L and 14 mmol/L stock solutions, respectively, in DMSO and stored at −80°C. Cultured cells were treated for 72 hours with a medium changes and fresh drug additions as indicated in the figure legends.

### Growth analyses

### Cell viability assay

Cells were seeded in triplicate at 5000 cells/well into 96-well plates, cultured with fresh growth medium for at least 24 hours and treated with a range of increasing concentrations of drugs for 72 hours. After the incubation period, 2-deoxyglucose (2-DG) and 3–4,5-dimethylthiazol-2-yl)-2,5-diphenyltetrazolium bromide (MTT, Sigma-Aldrich, St. Quentin Fallavier, France) were immediately added to the wells at a final concentration of 0.5 mg/mL, and the cells were incubated for 3 hours. Then, the supernatant was discarded, 100 μL of dimethyl sulfoxide (DMSO, Sigma-Aldrich, St Quentin Fallavier, France) was added, and the absorbance was monitored using a Flexstation 3 Plate reader (Sunnyvale, CA, USA) at 570 nm with 630 nm as a reference. The half maximal inhibitory concentration (IC_50_) was calculated with GraphPad Prism software version 5.00 for Windows (GraphPad Software, La Jolla, CA, USA). Each experiment was repeated at least 3 times.

### Clonogenic cell survival assay

After cell trypsinization with 1 mL trypsin-EDTA (Sigma-Aldrich Life Technologies, Saint Louis, MO, USA) and inactivation with medium containing 10% FBS, cells were seeded in triplicate at 150–200 cells per well into 6-well plates. The clones were allowed to grow for 15 days in growth medium with specific drugs at 37°C in an environment containing 5% CO_2_. The number of cell colonies was counted after fixing with 70% ethanol and staining with crystal violet. Data are represented as the mean ± SEM percentage values.

### Cell apoptosis assay

STS (soft-tissue sarcoma) cells (2 × 10^5^/well) were seeded in 6-well plates and treated for 72 hours with several specific drug concentrations. After treatment, cells were washed once with phosphate-buffered saline (PBS) and labeled with annexin-V-FITC and propidium iodide (PI) according to the manufacturer's protocol (BD Biosciences, San Jose, CA, USA). Then, cells were analyzed with a FACS (fluorescent activating cell sorting) Calibur flow cytometer (BD Biosciences, San Jose, CA, USA). The percentage of cells in early apoptosis (annexin-V-positive, PI-negative) and in late apoptosis or necrosis (annexin-V- and PI-positive) was calculated using FlowJo version 7.6.3 for Windows (Tree Star Inc, Ashland, OR, USA). The percentages of overall death (sum of early and late apoptosis) are represented as the mean ± SEM values based on 3 independent experiments.

### Western blotting

Treated and control whole cells were harvested using 60 μL radio-immunoprecipitation assay (RIPA) lysis buffer [35]. The lysate was centrifuged (13 000 rpm, 15 min, 4°C), and the supernatant was stored at −80°C until further use. Total proteins (30 μg) were electrophoresed on an 8, 12 or 15% sodium dodecyl sulfate (SDS) polyacrylamide gel and transferred onto polyvinylidene difluoride (PVDF) membranes. Blots were probed overnight at 4°C in 5% BSA (bovine serum albumin) in PBST (phosphate, 100 mM; KCl, 27 mM; NaCl, 1.37 M, pH 7.4 after 1X dilution; 0.1% Tween-20) with primary antibodies (diluted 1:1000) to p-AKT^ser473^ (CST 4060), AKT (CST 4685), p-S6RP^ser240/244^ (CST 2215), S6RP (CST 2217), p-ERK1/2^thr202/tyr204^ (CST 4370), ERK1/2 (ab17942), RICTOR (CST 2114) and glyceraldehyde-3-phosphate dehydrogenase (GAPDH, SC-51907). Horseradish peroxidase-conjugated secondary antibody (Santa Cruz Biotechnology, Inc., Heidelberg, Germany) was diluted 1:5000. Bound antibodies were visualized by Fusion Fx7 (Fisher Bioblock Scientific, Waltham, MA, USA) using Immobilon^TM^ Western (Millipore Corporation, Billerica, MA, USA), an enhanced chemiluminescence detection kit. The resulting bands were analyzed and quantified by ImageJ^®^ 1.49 g software (National Institutes of Health, Bethesda, MD, USA). GAPDH served as a loading control. Each membrane was reused twice after stripping in glycine buffer (6.6 mol/L, pH 2) at 56°C for 20 min. Each experiment was repeated at least 2 times.

### Ribonucleic acid interference (RNAi)

For transfections, 1 × 10^5^ LMS cells were seeded in duplicate into 6-well plates and grown to 60–70% confluency. Cells were transfected and incubated for 72 hours with 80 pmol RICTOR RNAi (AM16708-261571, Life Technologies, Carlsbad, CA, USA) using 6 μL Lipofectamine RNAiMAX (Invitrogen, Carlsbad, CA, USA) in a total volume of 300 μL growth medium containing 10% FBS. Then, protein samples were collected for western blotting. Each RNAi experiment was performed at least 2 times. Negative control (AM4613, Life Technologies, Carlsbad, CA, USA) using an RNAi that was not homologous to any known genes was used to control against nonspecific effects of the oligonucleotides.

### Drug synergy assays

LMS cells were treated with single drugs or a combination of two drugs for 72 hours. To confirm the synergistic effects between two drugs, a diagonal constant ratio combination design was implemented according to the Chou and Talalay proposition [22]. Cells were incubated with a 2-fold serial dilution with several concentrations above and below the IC_50_ values of both drugs at a constant ratio. After the incubation period, MTT was immediately added to the wells, and the absorbance was monitored using the Flexstation 3 Plate reader. The analysis of the synergy assay was conducted by the isobologram and combination index (CI) methods derived from the median-effect principle of Chou and Talalay. The combination effects of the two agents can be summarized as follows: combination index (CI) < 1 (under the curve), CI = 1 (near the curve), and CI > 1 (above the curve) indicates synergistic, additive and antagonistic effects, respectively. Synergy experiments were repeated at least three times.

### Animal studies

All animal experiments under project license DIR1384 were performed with the approval of the institutional animal use and care committee. LMS cells (5 × 10^6^ cells/100 μL) were injected subcutaneously into the right flank of Ragγ2C−/− mice (*n* = 10). Only the IB136 LMS cell line was able to grow in mice. Once palpable, the tumor volumes were calculated using the following formula: length × width^2^ / 2. After tumors reached approximately 100 mm^3^ in average size, animals were treated by oral gavage (100 μL). BEZ235 and GSK1120212 were prepared by dissolving in 1 volume of NMP (1-methyl-2-pyrrolidone) in a 100°C water bath followed by the addition of 9 volumes of PEG300 (Sigma-Aldrich, St Quentin Fallavier, France). GDC-0941 and everolimus were dissolved in water with 0.5% methylcellulose and 0.5% Tween 80 (Sigma-Aldrich, St Quentin Fallavier, France). To study of the effect of PI3K/AKT/mTOR pathway inhibitors on LMS tumor growth, mice were randomly assigned to receive 40 mg/kg BEZ235, 50 mg/kg GDC-0941, 5 mg/kg everolimus or the corresponding vehicle (NMP/PEG300 or methylcellulose). To study the anti-tumor effect of BEZ235 and GSK1120212 alone or in combination, mice were randomly treated with vehicle, 10 mg/kg BEZ235, 0.5 mg/kg GSK1120212 or a combination of both drugs. Drug concentrations were not the same as the previous *in vitro* experiments due to the strong synergy observed with the drug combination. Three weeks after drug administration, mice were euthanized, and tumors were excised. Tumor progression was analyzed with GraphPad Prism software and Kaplan-Meier curve analysis was used to compare individual tumor doubling rates. Log-rank (Mantel-Cox) tests were used to compare Kaplan-Meier curves with the program GraphPad Prism and *p*-values of 0.05 and below were considered significant.

### Immunohistochemistry

To perform immunohistochemistry on LMS cell line pellets, cells were grown to confluency in 6-well plates. Then, the cells were harvested, centrifuged at 1200 rpm for 5 minutes, incubated overnight in 10% paraformaldehyde and placed carefully into a cassette lined with biopsy filter paper. Next, the cell pellets were embedded in paraffin. Finally, sections of patient tumor samples and corresponding LMS cell line pellets were incubated with either anti-p-S6RP^ser240/244^ (CST 5364; 1:100) or anti-PTEN (CST 9559; 1:100). To confirm a negative labeling in tissues, we used endothelial cells as a positive control. For the *in vivo* study, three weeks after treatment with drugs or vehicles, mice were euthanized, and tumors were harvested in 10% paraformaldehyde. Paraffin sections were incubated with anti-Ki-67 (Ventana 790-4286; 1:100) and anti-p-S6RP^ser240/244^. Tissue imaging was conducted with an Olympus CKX41 (×100) using image capture cellSens Entry software version 1.14 for Windows (Olympus, Rungis, France). The immunoreactivity signal corresponding to the target expression level was estimated by a pathologist on the basis of the percentage of positively stained cells.

## SUPPLEMENTARY MATERIALS FIGURES


