# Supplementary material for: Dual inhibition of the PI3K/AKT/mTOR pathway suppresses the growth of leiomyosarcomas but leads to ERK activation through mTORC2: biological and clinical implications
Source: Oncotarget. 2016 Dec 16;8(5):7878–90. doi: 10.18632/oncotarget.13987 (PMC5352367; doi:10.18632/oncotarget.13987)
Supplement: Supplementary file 1 [file oncotarget-08-7878-s001.pdf]

Dual inhibition of the PI3K/AKT/mTOR pathway suppresses the growth of leiomyosarcomas but leads to ERK activation through mTORC2: biological and clinical implications

Supplementary Materials

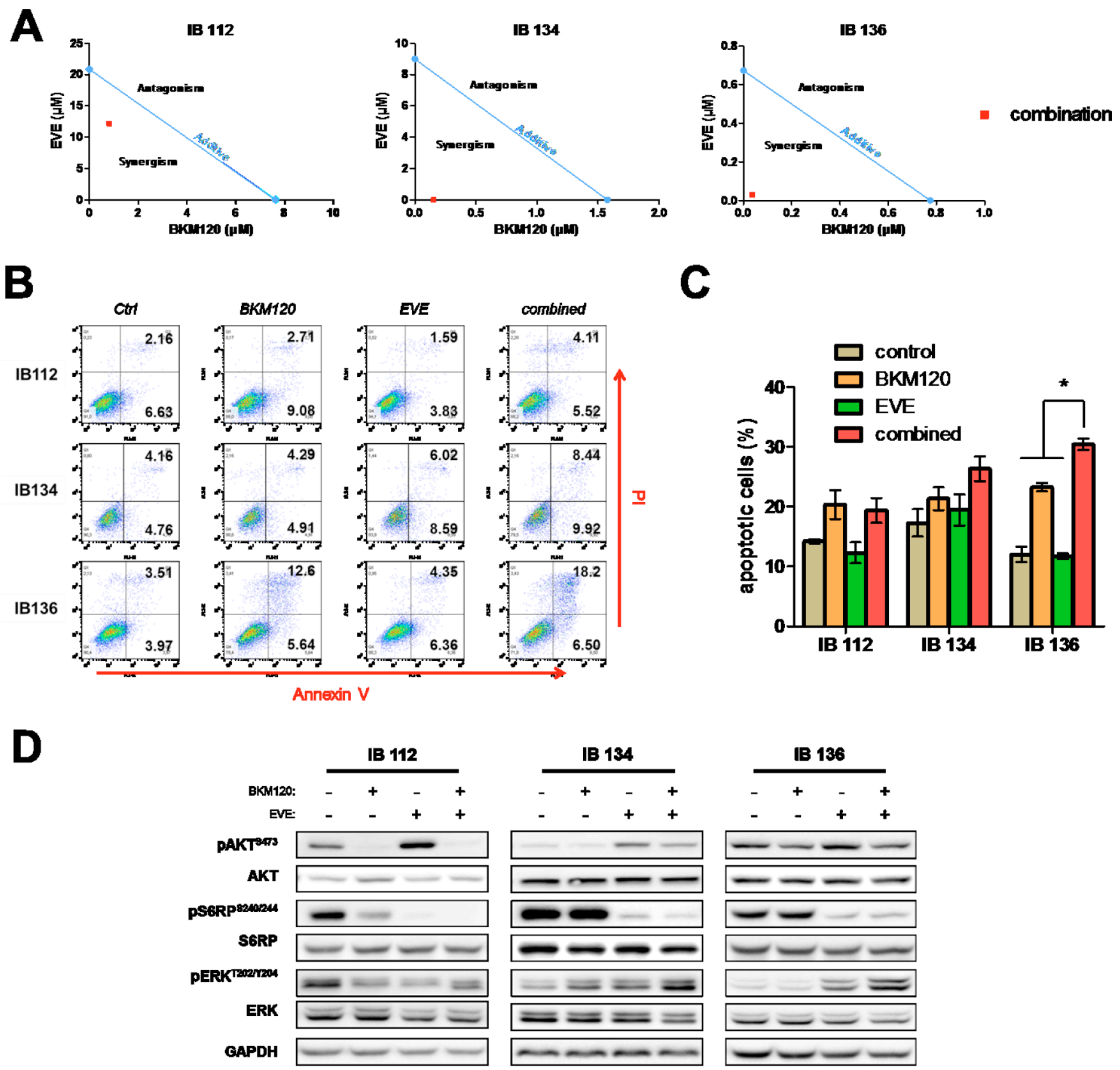

Supplementary Figure S1: Synergistic activity of BKM120 and everolimus on proliferation, apoptosis and PI3K and MAPK downstream signaling pathways in IB112, 134 and 136 LMS cell lines. Isobologram analysis of the combination of BKM120 and everolimus in LMS cells (**A**). Combination index (CI) values for each cell line were calculated using the method of Chou and Talalay and are represented by a red point. Representative dot-plot diagrams of flow cytometry with Annexin V/PI for LMS cells treated with BKM120 and EVE alone or in combination (**B**). Proportion of apoptotic cells after treatments (**C**). Immunoblotting analysis of active kinase and total kinase levels of PI3K/mTOR and MAPK pathway with GAPDH as a loading control (**D**). Cells were treated for 72 hours at IC50 value of each drug. Data presented are mean ± SEM of three independent experiments. *$p < 0.05$, two way ANOVA.

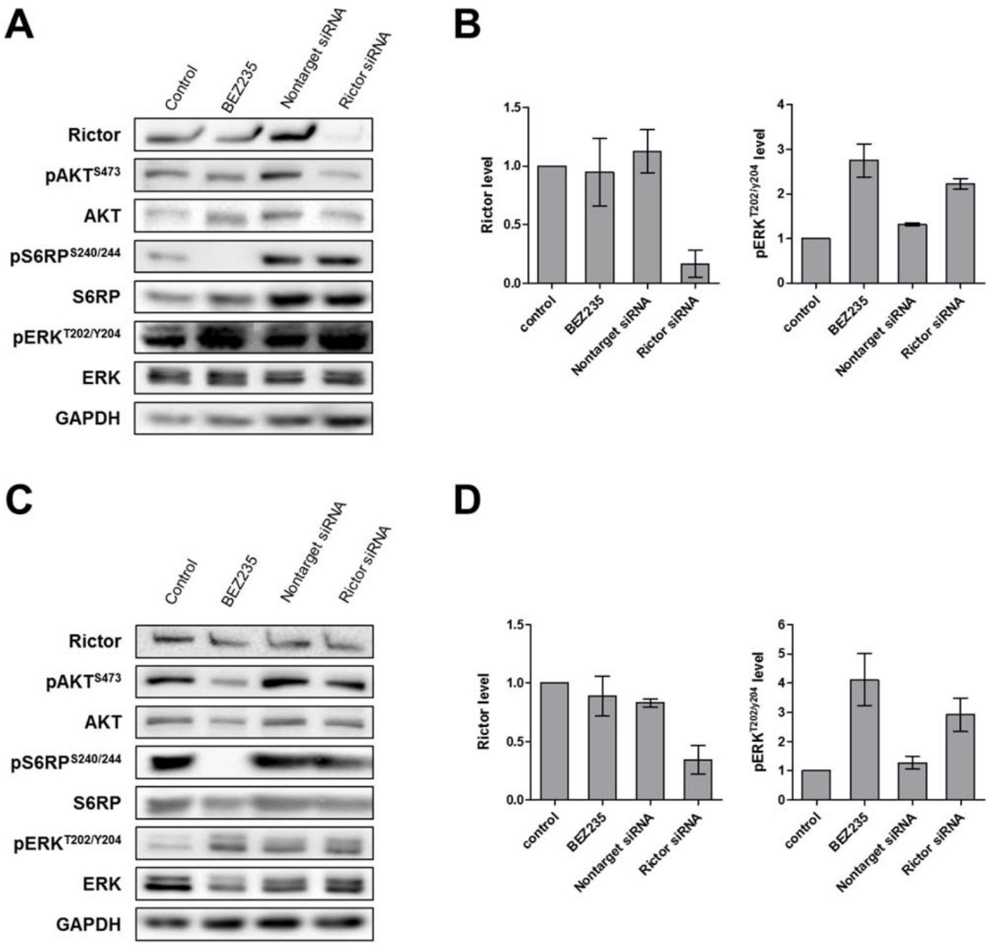

Supplementary Figure S2: Effect of RICTOR silencing on the PI3K and MAPK downstream signaling pathway in IB112 and 136 LMS cell lines. Representative western blotting of active kinase and total kinase levels of PI3K/mTOR and MAPK pathway with GAPDH as a loading control in IB112 (**A**) and IB136 (**C**) cell lines. LMS cell lines were incubated with BEZ235 and RNAi of RICTOR for 72 hours. Representation of signal intensities for RICTOR and p-ERK1/$2^{thr202/tyr204}$ were respectively normalized to those for GAPDH and ERK in IB112 (**B**) and IB136 (**D**) cell lines. Data presented are mean ± SEM of two independent experiments.
